# Vascularization of Engineered Spatially Patterned Myocardial Tissue Derived From Human Pluripotent Stem Cells *in vivo*

**DOI:** 10.3389/fbioe.2019.00208

**Published:** 2019-09-03

**Authors:** Maureen Wanjare, Masashi Kawamura, Caroline Hu, Cynthia Alcazar, Hanjay Wang, Y. Joseph Woo, Ngan F. Huang

**Affiliations:** ^1^Center for Tissue Regeneration, Repair and Restoration, Veterans Affairs Palo Alto Health Care System, Palo Alto, CA, United States; ^2^Stanford Cardiovascular Institute, Stanford University, Stanford, CA, United States; ^3^Department of Cardiothoracic Surgery, Stanford University, Stanford, CA, United States; ^4^Department of Bioengineering, Stanford University, Stanford, CA, United States

**Keywords:** cardiovascular tissue engineering, anisotropy, spatial patterning, pre-vascularization, electrospinning, epicardial patch, subcutaneous, vascular patterning

## Abstract

Tissue engineering approaches to regenerate myocardial tissue after disease or injury is promising. Integration with the host vasculature is critical to the survival and therapeutic efficacy of engineered myocardial tissues. To create more physiologically oriented engineered myocardial tissue with organized cellular arrangements and endothelial interactions, randomly oriented or parallel-aligned microfibrous polycaprolactone scaffolds were seeded with human pluripotent stem cell-derived cardiomyocytes (iCMs) and/or endothelial cells (iECs). The resultant engineered myocardial tissues were assessed in a subcutaneous transplantation model and in a myocardial injury model to evaluate the effect of scaffold anisotropy and endothelial interactions on vascular integration of the engineered myocardial tissue. Here we demonstrated that engineered myocardial tissue composed of randomly oriented scaffolds seeded with iECs promoted the survival of iECs for up to 14 days. However, engineered myocardial tissue composed of aligned scaffolds preferentially guided the organization of host capillaries along the direction of the microfibers. In a myocardial injury model, epicardially transplanted engineered myocardial tissues composed of randomly oriented scaffolds seeded with iCMs augmented microvessel formation leading to a significantly higher arteriole density after 4 weeks, compared to engineered tissues derived from aligned scaffolds. These findings that the scaffold microtopography imparts differential effect on revascularization, in which randomly oriented scaffolds promote pro-survival and pro-angiogenic effects, and aligned scaffolds direct the formation of anisotropic vessels. These findings suggest a dominant role of scaffold topography over endothelial co-culture in modulating cellular survival, vascularization, and microvessel architecture.

## Introduction

Cardiovascular disease is the leading cause of death in the US, with over 16 million people suffering from coronary heart disease and 0.7 million new myocardial infarcts per year (Benjamin et al., 2018). Clinical trials to restore cardiac function by therapeutic cell delivery have generally shown minimal to moderate benefit in improving cardiac pumping capacity (Dimmeler et al., 2008). As an alternative to cell transplantation, tissue engineering is a promising approach to regenerate myocardial tissue and to induce neovascularization. In particular, an important component of engineered tissues is the extracellular matrix (ECM), a scaffolding structure that provides signaling cues to cells through properties such as spatial patterning, stiffness, and cell binding domains.

It is well-established that the myocardium has highly organized physiological structure and ordered cellular orientation. However, the cells transplanted into the myocardium by mere injection are disarrayed in organization do not resemble the physiological orientation of native cardiac tissue. Consequently, to better reflect native cellular organization, parallel-aligned (anisotropic) scaffolds have been capable of providing provide spatial guidance cues that direct cellular rearrangement. Some of the approaches to fabricate parallel-aligned scaffolds include mechanical loading (Sellaro et al., 2007), microfluidic alignment (Lee et al., 2006; Lanfer et al., 2008), magnetic fields (Torbet and Ronziere, 1984; Guido and Tranquillo, 1993), soft lithography (Salick et al., 2014), and spatially oriented electrospinning (Zhong et al., 2006). Cardiomyocytes (CMs) seeded on such spatially patterned scaffolds have been shown to reorient their cytoskeleton along the direction of spatial patterning (Zong et al., 2005; Parrag et al., 2012; Wanjare et al., 2017; Allen et al., 2019). Among the various spatial patterning techniques, electrospinning is easily amenable to the fabrication of three-dimensional scaffolds over large areas with controllable nano-to-micro fibrous structure.

Besides spatially patterned biomaterials, another important consideration in the design of engineered myocardial tissue is the creation of organized microvasculature. Owing to the high metabolic rate of the myocardium, a continuous supply of blood from capillary networks that run parallel and adjacent to cardiac muscle fibers is required for sustained viability (Parker and Ingber, 2007). These capillary networks are composed of endothelial cells and mural cells. Integration with the host vasculature is critical to the survival and therapeutic efficacy of the engineered myocardial tissue. Consequently, cellular interaction between CMs and vascular endothelial cells (ECs) may be important for promoting integration with host microvasculature (Sekine et al., 2008; Huang et al., 2018; Zamani et al., 2018). Additionally, the presence of ECs within engineered myocardial tissues may promote angiogenesis as part of therapeutic remodeling after myocardial injury (Sekine et al., 2008; Gao et al., 2018).

In light of recent advancements in the directed differentiation of human pluripotent stem cells, it is now feasible to generate stem cell-derived CMs (iCMs) and ECs (iECs) from embryonic stem cell or induced pluripotent stem cell at high efficiency for cardiovascular tissue engineering (Burridge et al., 2014; Lian et al., 2014). The iCMs and iECs appear to mimic many phenotypic and functional capacities as native cells, and have been tested in animal models of cardiac or vascular regeneration (Rufaihah et al., 2011; Nakayama et al., 2018; Ishida et al., 2019). Consequently, iCMs and iECs are promising therapeutic cells for engineering of spatially patterned myocardial tissue.

Vascularization is critical to the survival of the engineered myocardial tissue as well as for promoting myocardial regeneration. Accordingly, the goal of this study was to determine the roles of scaffold anisotropy and endothelial interactions with iCMs on vascular integration of human pluripotent stem cell-derived engineered myocardial tissues *in vivo*. Randomly oriented or aligned scaffolds were seeded with iCMs and/or iECs to form engineered myocardial tissues. We then assessed the contribution of spatially patterned scaffolds and endothelial interaction in promoting tissue vascularization and the vascular network formation in both subcutaneous and myocardial injury models.

## Methods

### Polymer Preparation and Electrospinning

Electrospun microfibrous polycaprolactone (PCL) scaffolds were fabricated by electrospinning as previously described (Wanjare et al., 2017). Briefly, PCL (30% weight/volume PCL, Sigma Aldrich, M_n_ 80,000) and PEO (10% weight/volume, Sigma Aldrich, M_v_ 100,000) were simultaneously electrospun from separate syringes at 3.5 ml/h and 1.0 ml/h. Both polymers were co-electrospun using an electrospinner (Nanospinner 24-XP, Inovenso Ltd.) at a voltage of 25.0 kV, mandrel speed of 200 rpm, and a distance from spinneret to mandrel of 100 mm. Aligned scaffolds was generated from the randomly oriented scaffold using heat-based mechanical rearrangement of fiber organization, where segments of the scaffold sheet (12 × 25 × 0.1 mm) were stretched uniaxially to 300% of initial length using a 1 kg weight for 1 h at 55°C. Circular discs were cut from electrospun scaffold sheets using either a 6 mm or a 12 mm biopsy punch (Medex Supply). The scaffold discs were submerged in double distilled water at 37°C overnight with agitation to dissolve and remove the PEO from PCL. The scaffold discs were disinfected in 70% ethanol with agitation for 18 h. The scaffolds were then coated with Geltrex basement membrane ECM proteins (Sigma, 1:200 dilution) with agitation for 18 h to facilitate cell attachment onto the scaffolds.

### Microfiber Topography Imaging by Confocal Microscopy

Acellular PCL scaffolds with randomly oriented or aligned microfibrous structure were characterized for structural and mechanical properties after the PEO removal. Topographical analysis of the randomly oriented and the aligned fibrous scaffolds was performed using confocal microscopy at an emission wavelength of 460–490 nm. Fibers were visible at these wavelengths owing to the intrinsic fluorescence of the microfibers in this emission window. Using the confocal microscopy images obtained from randomly oriented and the aligned fibrous acellular scaffolds, the fiber orientation was quantified using the two-dimensional Fast Fourier Transform (FFT) tool in ImageJ.

### Scaffold Degradation Assay

Lipase, a water-soluble enzyme that hydrolyzes ester bonds of water-soluble substrates, has been shown to degrade PCL (Zeng et al., 2004; Ferreira et al., 2017). To assess the kinetics of PCL degradation, 6-mm circular discs of PCL scaffolds were subsequently washed with distilled water for 24 h to remove sacrificial PEO polymer. Following the drying of scaffolds for 24 h, scaffolds were immersed scaffolds 0.5 mg/ml lipase enzyme solution (from *Burkholderia cepacia*, Sigma) and incubated at 37°C for up to 14 days. As a control for enzyme-mediated degradation, separate samples were included in phosphate buffered saline. At specified intervals (days 1, 4, 8, 12, and 14), the scaffolds were taken out the lipase enzyme solution and placed in distilled water for 24 h. The lipase enzyme solution was then replaced with fresh enzyme solution at each interval for the duration of the experiments. At each interval, the scaffolds were then dried for 24 h and weighed at room temperature. The scaffold weight loss percentage was calculated below (*n* = 5), where *W*_0_ denotes the original weight and *W*_*T*_ denotes weight at the designated time interval:

(1) $$\begin{array}{r} Scaffold\;remaining\;=\left(\frac{W_{T}}{W_{0}}\right)\;\times 100 \end{array}$$

### Human Pluripotent Stem Cell Culture

The human induced pluripotent stem cell line (P356) was generated by reprogramming of healthy human peripheral blood mononuclear cells using Sendai virus-mediated transduction of Sox2, Oct3/4, KLF4, and c-myc (Carcamo-Orive et al., 2017). This line was used for subcutaneous implantation studies. The human embryonic stem cell line (H9, WiCell Research Institute, Madison, WI) was used for epicardial transplantation studies. Both lines were passaged every 4 days using ethylenediaminetetraacetic acid (0.1 mM, EDTA) dissociation reagent (Invitrogen). The PSC cells were expanded on tissue culture dishes coated with Geltrex basement membrane matrix extract (1:200 dilution, Sigma) in Essential 8 expansion medium (Invitrogen). All cells were maintained in humidified incubators at 37°C and 5% CO_2_.

### Generation of iCMs

Human pluripotent stem cells were differentiated into iCMs using an established differentiation procedure as previously described (Wanjare et al., 2017). Briefly, iCMs were generated using a directed differentiation method that involved incubating iPSCs with Wnt agonist CHIR 99021 (6 μM, Selleck), followed by Wnt antagonist IWR-1 (5 μM, Selleck) in Roswell Park Memorial Institute (RPMI) 1640 medium (Thermo Fisher), supplemented with insulin-free B27 (Invitrogen). After 7 days, the culture medium was replaced with RPMI 1640 supplemented with B27 and insulin (Invitrogen). To enrich the iCM populations, the differentiating cells were deprived of glucose after 9 days. The iCMs were used for experiments after 15 days of differentiation. Only dishes containing >80% spontaneously contracting iCMs were used for the described experiments.

### Generation of iECs

Induction of endothelial differentiation was carried out using an established protocol (Wu et al., 2015; Wanjare et al., 2017). Briefly, the iPSCs were supplemented with Wnt agonist CHIR 99021 (5 μM, Selleck), bone morphogenetic protein-4 (25 ng/mL, Peprotech), B27 supplement (Gibco), and N2 supplement (Gibco). After 3 days, the cells were dissociated with HyQtase (Fisher Scientific) and plated at a density of 3.3 × 10^4^ cells/cm^2^ in StemPro media (Gibco), supplemented with forskolin (5 μM, LC Labs), vascular endothelial growth factor (VEGF, 50 ng/mL, Peprotech), and polyvinyl alcohol (2 mg/mL, Sigma). After 7 days, the cells were washed twice with PBS, and then cultured in endothelial growth media (EGM-2MV, Lonza) supplemented with additional VEGF (100 ng/ml) for 7 more days. For indicated *in vivo* experiments, the human iECs derived from induced pluripotent stem cells were further lentivirally transduced between day 11–14 of differentiation using a double fusion reporter construct consisting of the ubiquitin promoter driving firefly luciferase (luc) and green fluorescence protein (GFP) (Huang et al., 2010; Nakayama et al., 2015).

### Cell Seeding in Scaffolds

Scaffolds were seeded as previously described (Wanjare et al., 2017). In brief, scaffolds (6-mm in diameter) were seeded into one face of the scaffold, while 12-mm scaffolds were seeded on both sides of the scaffolds. Singular cultures of iCMs were generated by dissociating iCMs from tissue culture dishes after differentiation using TrypLE 10X (ThermoFisher) (Gibco) and then seeding into the randomly oriented or aligned scaffold groups at a density of 1 × 10^6^ cells per scaffold in iCM culture medium consisting of RPMI 1640 containing B27 and insulin. Singular cultures of iECs were generated by dissociating iECs from tissue culture dishes after differentiation using HyQtase and seeding of 4 × 10^4^ cells onto scaffolds. Co-culture treatment groups were seeded with both iCMs and iECs, in which the cells were seeded in a sequential fashion. First, iECs were dissociated from cell culture dishes using HyQtase and seeded onto the scaffolds at a density of 4 × 10^4^ per scaffold in EGM-2MV endothelial expansion media. After 3 days, iCMs were then seeded onto either randomly oriented or aligned scaffolds at a density of 10^6^ cells per scaffold in iCM culture medium. The cell-seeded scaffolds were harvested 2 days later for *in vivo* transplantation.

### *In vitro* Characterization of Engineered Myocardial Tissue by Immunofluorescence Staining

Immunofluorescence staining was performed after cell seeding to visualize the *in vitro* organization of human iCMs and iECs within the scaffolds, based on phenotypic markers of troponin-T (TNNT) for iCMs and CD31 for iECs. The cell-seeded scaffolds were fixed in 4% paraformaldehyde (Electron Microscopy Sciences), permeabilized in 0.1% Triton X-100 (Sigma-Aldrich), and then blocked with 1% bovine serum albumin (Wanjare et al., 2017). The scaffolds were then incubated with primary antibodies directed against anti-human troponin-T (Sigma) and anti-human CD31 (Dako), followed by secondary antibodies conjugated to Alexa Fluor 488 or Alexa Fluor 594 (both from Thermo Fisher Scientific). The samples were then washed in PBS, and total nuclei were counterstained using Hoechst 33342 (Thermo Fisher Scientific) nuclear dye. Samples were stored at 4°C in the dark and imaged using a laser scanning confocal microscope (LSM710, Zeiss).

### Murine Subcutaneous Transplantation of Engineered Myocardial Tissue

NOD SCID mice (male, 8–10 weeks old, Jackson Labs) were used in subcutaneous implantation studies. The mice were anesthetized with 2% isoflurane (Fluriso^TM^, VetOne), and then placed in a prone position to expose the upper back. After disinfecting the skin of the mice using 70% ethanol and a betadine solution, incisions were made to the skin of the back and expanded into subcutaneous pockets using sterile cotton swabs. One of the following cardiac engineered myocardial tissues was inserted into each pocket: (1) randomly oriented scaffold seeded with iCMs; (2) randomly oriented scaffold seeded with iECs; (3) randomly oriented scaffold seeded with iCMs + iECs; (4) aligned scaffold seeded with iCMs; (5) aligned scaffold seeded with iECs; (6) aligned scaffolds seeded with iCMs + iECs; (7) acellular randomly oriented scaffold; or (8) acellular aligned scaffold (*n* ≥ 3). After engineered myocardial tissue transplantation, 5–0 sutures were used to close the pockets. Slow releasing buprenorphine (1 mg/kg) was given subcutaneously for analgesia.

For non-invasive imaging of cell survival *in vivo* in a subset of the animals, iECs within cell-seeded scaffolds were lentivirally transduced to express firefly luciferase, as described previously (Huang et al., 2010; Nakayama et al., 2015; Foster et al., 2018; Zaitseva et al., 2019). The iECs used for bioluminescence imaging were all derived from the same batch of viral transduction for consistency. The parental pluripotent stem cells were not transduced for luciferase gene expression due to potential concerns of gene silencing during the differentiation process (Wilson et al., 2008; Lepperhof et al., 2014). At time points up to 2 weeks, the animals with luciferase-tagged cells underwent bioluminescence imaging. Animals were injected intraperitoneally with D-luciferin that catalyzes the release of photons that can be captured by an IVIS-200 (Xenogen) bioluminescence detection system. Since only living cells can catalyze the release of photons, this detection method selectively quantifies the relative number of viable cells. Bioluminescence intensity correlates linearly to viable cell numbers (Huang et al., 2010; Rufaihah et al., 2011). The bioluminescence signal was acquired in units of mean radiance (p/s/cm^2^/sr) and then expressed over time as the percent difference relative to day 0.

At 2 weeks after transplantation, the animals were euthanized, and a skin incision was made to reveal the subcutaneous scaffolds. The scaffolds within the subcutaneous pockets were explanted and processed for routine paraffin-embedding. *En face* tissue sections (5-μm-thick) were placed on silanized slides for immunohistochemistry or immunofluorescence staining. These studies were approved by the Institutional Animal Care and Use Committee at the Veterans Affairs Palo Alto Health Care System.

### Epicardial Transplantation of Engineered Myocardial Tissue Onto Rat Injured Myocardium

Immunocompromised RNU rats (male, 200–250 g, Charles River) were anesthetized with 2% isoflurane (Fluriso^TM^, VetOne), intubated with a 16G angiocatheter, and maintained on a ventilator (Hallowell EMC, Pittsfield, MA). The heart was exposed through a left thoracotomy, and the left coronary artery was permanently ligated using a 6-0 polypropylene suture placed 3 mm below the left atrial appendage to produce a relatively small anterolateral infarct. To ensure consistently-sized infarcts, the suture was snared prior to ligation and the area of resulting pallor was assessed. Immediately after induction of myocardial injury by arterial ligation, the animals were randomized to receive one of the following treatment groups: (1) randomly oriented scaffold seeded with iCMs; (2) randomly oriented scaffold seeded with iCMs + iECs; (3) aligned scaffold seeded with iCMs; (4) aligned scaffold seeded with iCMs + iECs; (5) acellular randomly oriented scaffold (*n* ≥ 3). The engineered myocardial tissues were secured to the left ventricular epicardium by a single suture. The thorax was then suture closed, and the animals were allowed to recover for 1 month. Echocardiography was performed pre-operatively, as well as post-operatively on days 4 and 28 for assessment of ejection fraction (Vevo 2100, Visual Sonics). Buprenorphine was provided for analgesia (0.1 mg/kg). After 1 month of transplantation, the rats were euthanized and the myocardium was explanted for routine paraffin embedding. Transverse tissue sections (5 μm-thick) were placed on silanized slides for routine hematoxylin and eosin (H&E) staining, immunohistochemistry and immunofluorescence staining. These studies were approved by the Institutional Animal Care and Use Committee at the Veterans Affairs Palo Alto Health Care System.

### Immunohistochemistry and Immunofluorescence Staining of *in vivo* Samples

Immunohistochemical staining was carried out using the DAKO Animal Research Kit perioxidase system (Agilent), according to the manufacturer's instructions (Huang et al., 2005). Following deparaffinization, the tissue sections were treated with low pH antigen retrieval solution (Thermo Fisher) for epitope recovery at 100°C for 20 min. Immunofluorescence staining was performed as described above to visualize host murine endothelial cells and smooth muscle cells based on CD31 (BD Transduction Labs) and smooth muscle α-actin (SMA, Sigma), respectively. The contractile proteins troponin-T and troponin-I (both from Abcam), were used to visualize human iCMs, and a human-specific CD31 antibody (Dako) was used to visualize human iECs. Human and mouse tissues were used as positive and negative controls, respectively, to confirm species specificity.

### Immunofluorescence Analysis of Host Revascularization and Vascular Organization

The mean capillary density of subcutaneously transplanted engineered myocardial tissues was quantified based on confocal microscopy images of *en face* tissue sections stained by murine-reactive CD31 antibody (BD Transduction Labs). Capillaries were identified as a single layer of CD31^+^ cells with flattened morphology. For each sample, four images for each sample were acquired using 20X objectives (*n* ≥ 3). From each image, the total number of capillaries was manually counted and expressed in the form of capillary density (#/mm^2^) (Rufaihah et al., 2011). The percentage of total capillaries with longitudinal or transverse orientation was further recorded. Within the *en face* tissue sections, the orientation of vessels was further categorized as being longitudinal, transverse, or of other orientations, and was expressed as a distribution. Among the longitudinally oriented vessels within the subcutaneously explanted engineered myocardial tissues, the orientation was quantified with respect to the axis of the aligned microfibers. From confocal microscopy images of CD31 staining, an ImageJ macro (FibrilTool) was used to quantify the global angle of vessel orientations from range of 0–90°, where 0° denotes parallel alignment relative to the axis of the aligned microfibers, and 90° denotes orthogonal orientation with respect to the aligned microfiber axis. For randomly oriented scaffolds, an arbitrary axis was selected. From tissue cross sections of engineered myocardial tissues that were transplanted onto the rat epicardium, arterioles within the engineered tissues were identified having visible lumen with a diameter of 10–100 μm and by the co-expression of SMA and CD31. The average number of arterioles out of four representative images was quantified per sample, and then expressed in the form of arteriole density (#/mm^2^) (Huang et al., 2005; Hadamitzky et al., 2016).

### Statistical Analysis

Data are shown as mean ± standard deviation. A Student's *t*-test was used for comparisons between two groups. One-way analysis of variance (ANOVA) with Bonferroni adjustment was performed for comparisons of three or more treatment groups. A repeated measures ANOVA with Bonferroni adjustment was used for analysis of the same treatment group at multiple time points. A value of *P* < 0.05 was considered statistically significant.

## Results

### Biophysical Characterization of Microfibrous 3D Scaffolds

**Figure 1:**
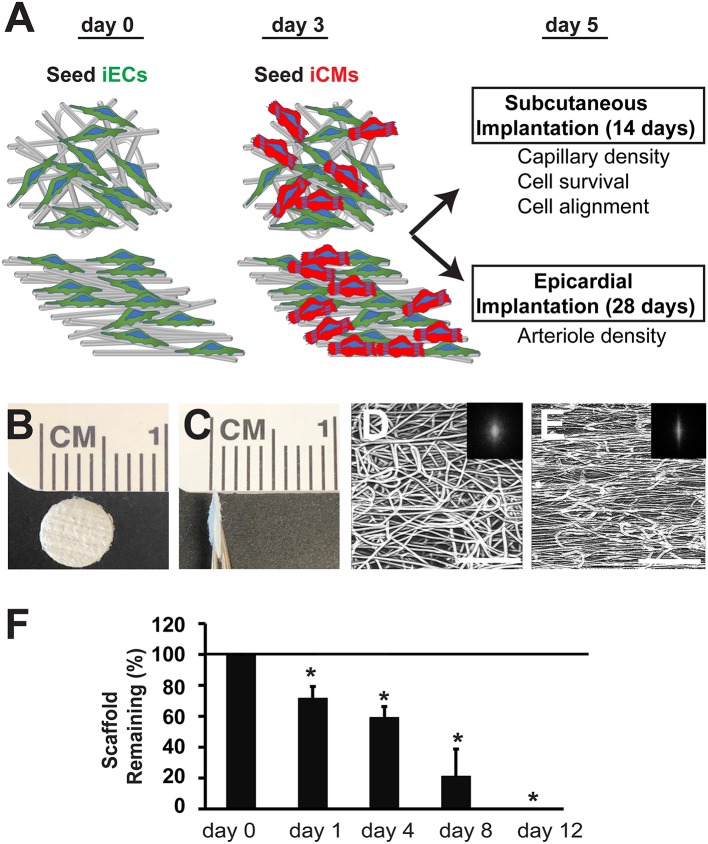
Biophysical characterization of microfibrous PCL scaffolds. **(A)** Schematic overview of experimental design depicts cell seeding of iECs and iCMs into randomly oriented or aligned microfibrous scaffolds for either subcutaneous or epicardial transplantation. **(B,C)** Gross morphology of electrospun PCL scaffolds is shown by *en face* **(B)** and cross-sectional **(C)** aspects. **(D,E)** Confocal microscopy images of microfiber organization based on autofluorescence. The alignment of the corresponding microfibers was quantified by two-dimensional Fast Fourier Transform analysis and is depicted by frequency plots in the insets. **(F)** Degradation of acellular scaffolds in lipase (*n* = 5). Horizontal line at 100% denotes degradation of scaffolds when incubated in saline only. Scale bar: 200 μm **(D,E)**. *Depicts statistically significant relationship (*P* < 0.05).

The electrospun scaffolds with parallel-aligned or randomly oriented microfiber organization were characterized by their spatial organization and degradation (Figure 1). Both aligned and randomly oriented scaffolds had similar cross-sectional thicknesses of ~400 μm and microfiber diameter of 7–14 μm (Figures 1B,C). However, the topography of the two kinds of scaffolds was distinctively different. The randomly oriented scaffolds were composed of microfibers with arbitrarily organization, whereas the aligned scaffolds consisted of microfibers arranged about a single axis (Figures 1D,E). These observations were confirmed by FFT analysis, in which the pixelsin the frequency plot of the aligned scaffold (Figure 1E, inset) depicted a principal angle of orientation about the central origin that is consistent with anisotropy, whereas the pixels in the frequency plot of the randomly oriented scaffold (Figure 1D, inset) lacked any principal angle of orientation and was consistent with of isotropy.

To characterize the PCL scaffolds based on their biodegradation kinetics, *in vitro* hydrolytic degradation studies were conducted in the presence of lipase enzyme (Khan et al., 2017). In the presence of lipase, PCL scaffold weight was measured in the dry state over time. The weight loss of PCL scaffolds over the course of 12 days was linearly correlated (*R*^2^ = 0.96), resulting in complete degradation of the scaffolds after 12 days (Figure 1F). However, scaffolds that were not exposed to lipase did not degrade and remained intact after 12 days in saline. This data suggests that the PCL scaffolds can undergo enzymatic degradation, but not in physiological saline conditions.

### *In vitro* Characterization of Engineered Myocardial Tissues

**Figure 2:**
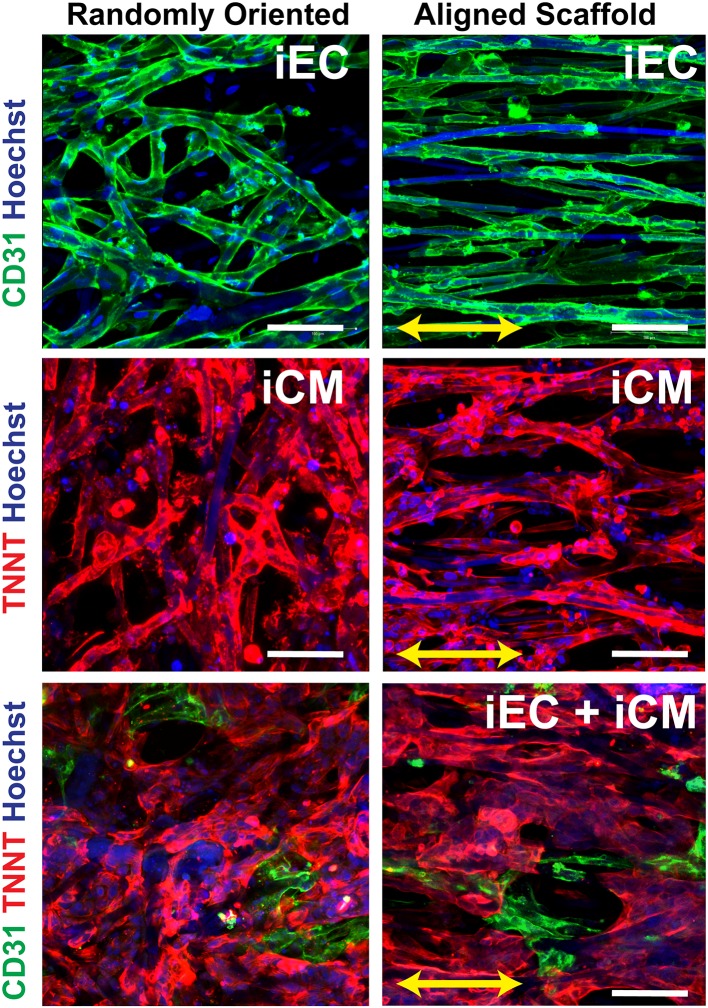
*In vitro* characterization of engineered myocardial tissue composed of iCMs and iECs cultured in aligned or randomly oriented microfibrous scaffolds. Representative confocal images depict the attachment and cellular organization of iECs after 3 days of seeding, followed by the co-culture with iCMs for 2 more days. The iCMs were visualized by troponin-T (TNNT) expression (red), and iECs were visualized by CD31expression (green). Total nuclei were depicted by Hoechst 33342 dye (blue). Arrow denotes the orientation of the microfibers on aligned scaffolds. Scale bar: 100 mm.

The iECs and iCMs derived from human induced pluripotent stem cells were seeded on Geltrex-coated scaffolds, as previously described (Wanjare et al., 2017). As shown in Figure 2, the iCMs were visualized by troponin-T cytoskeletal protein (red), whereas the iECs selectively expressed the cell adhesion marker, CD31 (green). The cellular attachment was robust after 3 days, without any indications of cytotoxicity. In engineered myocardial tissue composed of both iECs and iCMs, the iCMs dislodged some of the iECs after 2 days of co-culture, leading to a lower population of iECs, than compared to scaffolds comprising only iECs. The engineered myocardial tissue derived from randomly oriented scaffolds, regardless of whether consisting of iCMs only or iCMs+iECs, appeared to have an arbitrary cellular organization, similar to the disorganized appearance of the underlying microfiber structure (Figure 2). On the other hand, engineered myocardial tissue derived from aligned scaffolds appeared to undergo reorganization by aligning their cytoskelelton along the direction of the aligned microfibers (Figure 2). Both iECs and iCMs wrapped around single or multiple fibers in order to assume an elongated morphology along the direction of the aligned microfibers. These data suggest that engineered myocardial tissue derived from aligned scaffolds could support spatial patterning of both iCMs and iECs *in vitro*.

### Survival of Engineered Myocardial Tissue in a Murine Subcutaneous Transplantation Model

**Figure 3:**
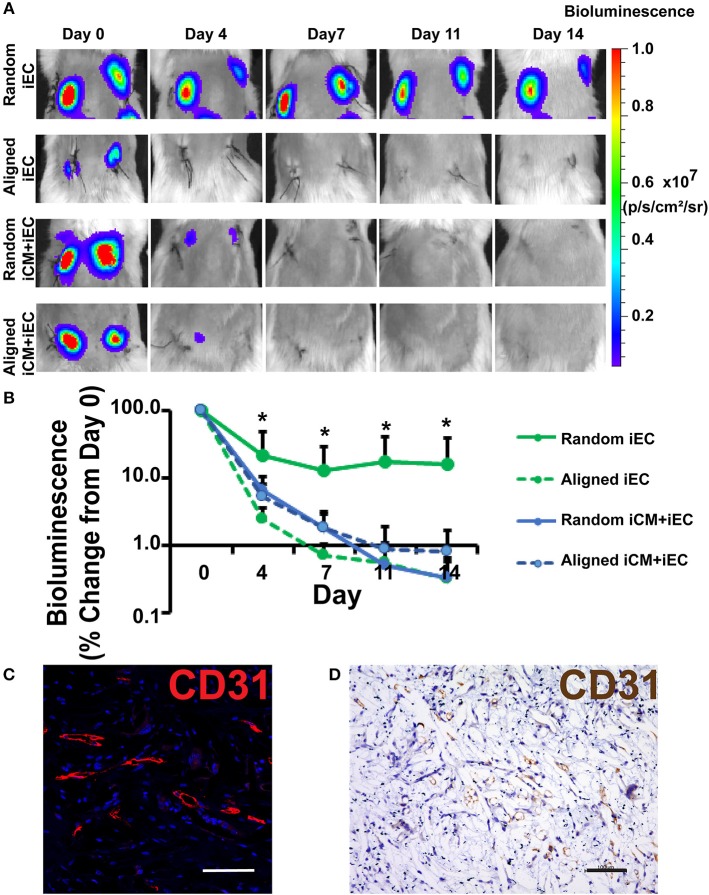
Non-invasive imaging of iEC survival following subcutaneous implantation of engineered myocardial tissue in mice. **(A)** Representative bioluminescence images of subcutaneously implanted engineered myocardial tissue cultured with luciferase-tagged iECs over the course of 14 days. Shown are duplicate engineered myocardial tissues implanted into the subcutaneous space of the same mouse. **(B)** Quantification of iEC survival *in vivo* based on bioluminescence imaging (*n* ≥ 4). Treatment groups consisted of engineered myocardial tissue composed of either randomly oriented or aligned scaffolds seeded with iECs alone or in co-culture with iCMs. **(C,D)** Visualization of iECs within explanted engineered myocardial tissue formed from randomly oriented scaffolds using a human-specific CD31 antibody by immunofluorescence staining (**C**, red) or immunohistochemical detection (**D**, brown). *Denotes statistically significant in comparison to all other treatment groups (*P* < 0.05). Scale bars: 100 μm **(C,D)**.

To test the efficacy of engineered myocardial tissue to support cell survival *in vivo*, the cell-seeded scaffolds were transplanted subcutaneously into NOD SCID mice for 14 days. The iECs were further lentivirally transduced to confer non-invasive bioluminescence imaging based on constitutive luciferase gene expression. Since luciferase expression was restricted to viable cells, bioluminescence imaging could be used to quantify the relative survival of iECs within the subcutaneously transplanted engineered myocardial tissues. Over the course of 14 days, the iEC bioluminescence signal showed a reduction in viability for all treatment groups (Figure 3A). After 4 days of transplantation, the randomly oriented scaffolds seeded with iECs alone demonstrated a significantly higher iEC survival (37 ± 30%), compared to aligned scaffolds seeded with iECs (2 ± 0.8%), aligned scaffolds seeded with iCMs+iECs (5 ± 4%), or randomly oriented scaffolds seeded with iCMs+iECs (6 ± 4%) (*P* < 0.05, *n* ≥ 4, Figure 3B). After 14 days of transplantation, the randomly oriented scaffolds seeded with iECs alone continued to promote a significantly higher iEC survival (22 ± 21%), compared to aligned scaffolds seeded with iECs (0.3 ± 0.3%), aligned scaffolds seeded with iCMs+iECs (0.9 ± 1.5%), or randomly oriented scaffolds seeded with iCMs + iECs (0.3 ± 0.2%) (*P* < 0.05, *n* ≥ 4). To verify the viability of iECs, the engineered myocardial tissues were explanted after 14 days for immunofluorescence and immunohistochemical staining of human specific CD31 expression (Figures 3C,D). Survival of the iCMs within engineered myocardial tissues was assessed by immunofluorescence staining of troponin-I. Histological analysis showed minimal expression of troponin-I in any treatment group, which suggested that the iCMs were no longer present after 14 days. Additionally, the PCL scaffolds did not show signs of marked degradation during explantation on day 14. These data suggest that randomly oriented scaffolds preferentially promoted the survival of iECs in a subcutaneous transplantation model.

### Organization of Host Microvasculature Within Engineered Myocardial Tissue in a Murine Subcutaneous Model

**Figure 4:**
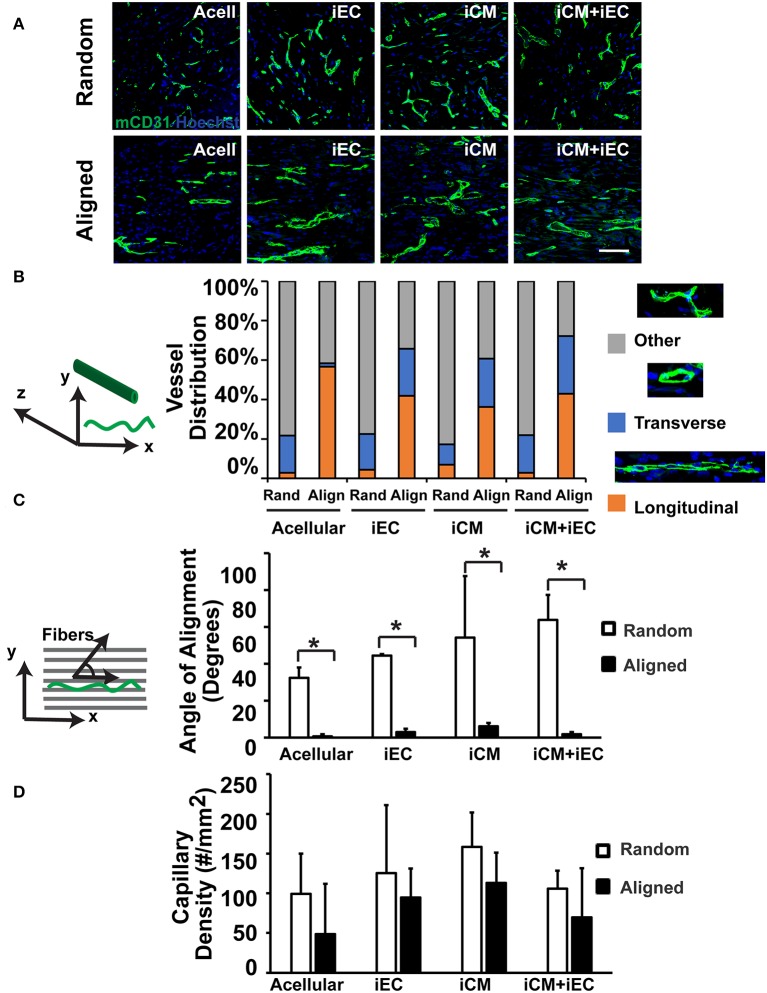
Vascularization of engineered myocardial tissue following subcutaneous implantation into mice. **(A)** Representative confocal microscopy images of CD31 staining (green) within *en face* sections of engineered myocardial tissues derived from randomly oriented or aligned nanofibrillar scaffolds containing iCMs, iECs, or iCM+iECs at 2 weeks after subcutaneous implantation. Acell denotes acellular scaffold. **(B)** Distribution of vessel orientations within explanted engineered myocardial tissue, relative to the axis of the aligned microfibers as longitudinal, transverse, or other. **(C)** Quantification of the global angle of vessel alignment within subcutaneously explanted engineered myocardial tissues, relative to the axis of the aligned microfibers. The global angle of vessel alignment is calculated as the angle formed by the direction of the longitudinally oriented vessel with respect to the axis of the aligned microfibers. For randomly oriented scaffolds, an arbitrary axis was selected (*n* ≥ 3). **(D)** Quantification of murine capillary density within the explanted engineered myocardial tissue (*n* ≥ 3). *Denotes statistically significant in comparison (*P* < 0.05).

To determine if scaffold anisotropy had differential effects on vascular regeneration, *en face* sections of the explanted engineered myocardial tissues were immunofluorescently stained for host capillaries using a murine-reactive CD31 antibody (Figure 4A). Intriguingly, the capillaries within the explanted engineered myocardial tissues showed distinctively different architectural organization. We categorized the organization of the capillaries as being longitudinal (capillaries arranged in the plane of the *en face* sections), transverse (capillaries organized transversely with respect to the *en face* sections), or other (capillaries with neither longitudinal nor transverse arrangement). Among the engineered myocardial tissue derived from aligned scaffolds, the percentages of total capillaries with longitudinally orientation were within the range of 36–56%, suggesting a relative high percentage of longitudinally oriented vessels (Figure 4B). In contrast, among engineered myocardial tissues derived from randomly oriented scaffolds, the percentage of total vessels with longitudinally oriented vessels were within the range of 3–7%. This data suggests that aligned scaffolds supported the formation of longitudinal vessels. Furthermore, among the longitudinally oriented vessels, we further quantified the alignment of capillaries, relative to the axis of the aligned microfibers (Figure 4C). The mean alignment of longitudinal capillaries within engineered myocardial tissues derived from randomly oriented scaffolds seeded with iECs (44 ± 0.8°), iCMs (54 ± 33°), or iECs+iCMs (63 ± 14°) was consistent with isotropic vessel arrangement that lacked any preferential orientation. In contrast, the alignment of capillaries within engineered myocardial tissues derived from aligned scaffolds seeded with iECs (3.0 ± 1.8°), iCMs (6.1 ± 1.8°), or iECs+iCMs (1.9 ± 1.2°) suggested that the aligned microfibers could preferentially guide the organization of host microvasculature along the direction of the aligned microfibers. Despite the difference in host microvasculature arrangement, however, the capillary density was similar between engineered myocardial tissues derived from aligned or randomly oriented scaffolds (Figure 4D). Together, this data demonstrated that engineered myocardial tissues derived from aligned scaffolds promoted the anisotropic arrangement of capillaries, resulting in the formation of longitudinal vessels aligned in close proximity along the direction of the microfibers.

### Host Vascularization of Engineered Myocardial Tissue Following Rat Epicardial Transplantation

**Figure 5:**
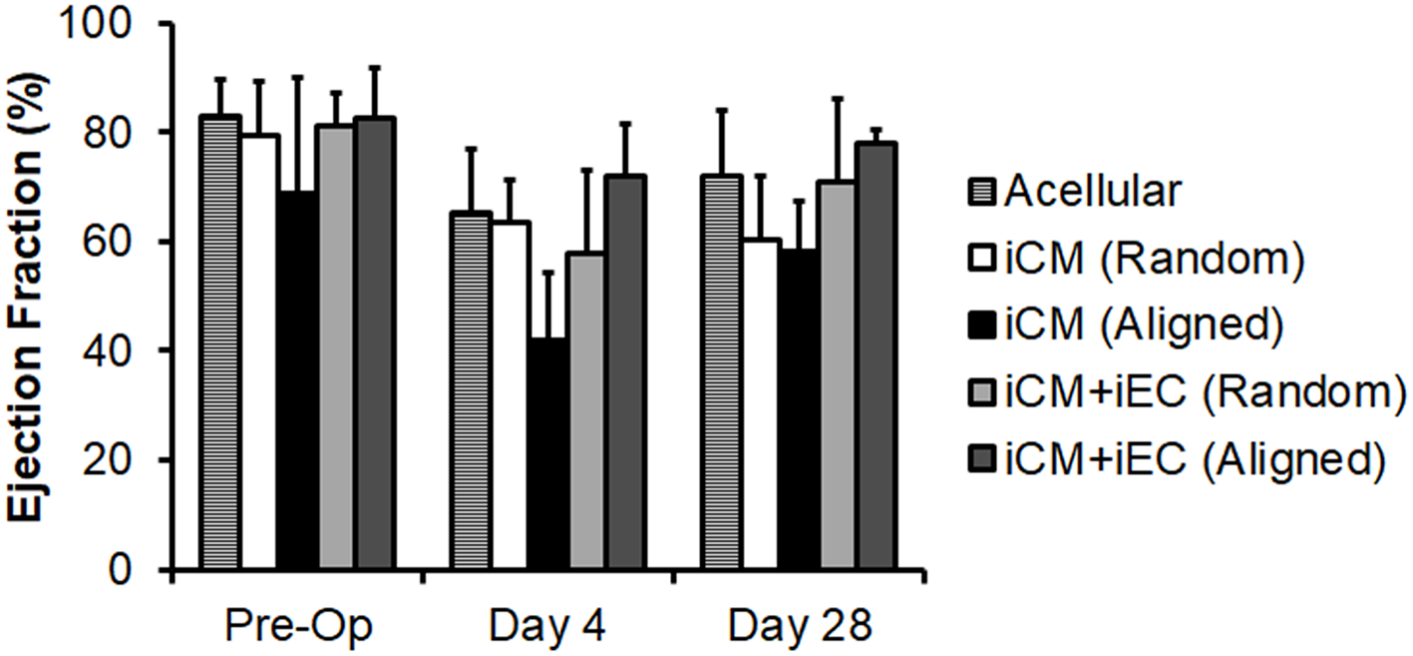
Quantification of ejection fraction following epicardial implantation onto injured rat myocardium. Treatment groups consisted of engineered myocardial tissues derived from randomly oriented or aligned nanofibrillar scaffolds containing iCMs or iCM+iECs at 4 weeks after epicardial transplantation. Acell denotes acellular scaffold. Shown are ejection fractions measured pre-operatively, as well as on days 4 and 28 after myocardial injury and treatment (*n* ≥ 3).

**Figure 6:**
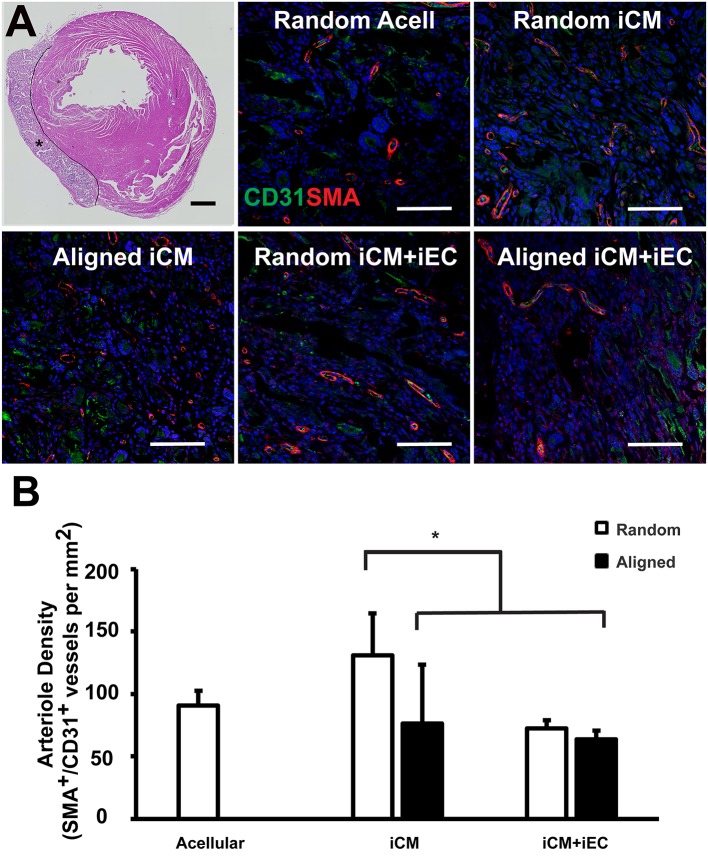
Vascularization of engineered myocardial tissue following epicardial implantation onto injured rat myocardium. **(A)** Representative hematoxylin and eosin staining (H&E) of transverse cross sections of the ventricles showing the location of the transplanted engineered myocardial tissue (denoted by *, with black line showing the boundaries of the engineered myocardial tissue) at 28 days after transplantation. Transverse tissue sections of explanted engineered myocardial tissue were immunofluorescently stained for murine-reactive antibodies against CD31 for endothelial cells (green) and smooth muscle α-actin (SMA, red). **(B)** Quantification of arteriole density within the explanted engineered myocardial tissues derived from aligned or randomly oriented scaffolds and cultured with iCM, iCM+iEC, or no cells (Acell). The microvessel density was calculated as the density of CD31^+^/SMA^+^ vessels per mm (*n* ≥ 3). *Denotes statistically significant relationship (*P* < 0.05). Scale bar: 1 mm (H&E); 100 μm (CD31/SMA).

Since the total capillary density was not significantly different between engineered myocardial tissue groups in a subcutaneous transplantation model, we reasoned that an injury model would be suitable for observing changes in host vascular integration. A mild myocardial injury model was employed to induce reparative mechanisms that could stimulate vascular integration, although not severe enough to markedly alter cardiac function. Mild myocardial injury was induced in rats by ligation of the coronary artery, followed by epicardial transplantation of the engineered myocardial tissues. To confirm the induction of mild ischemia, quantitative assessment of mean ejection fraction among the treatment groups showed a small decline in ejection fractions ranging from 68 to 83% pre-operatively to 42–72% on day 4 post-operatively (Figure 5). Compared to reported values of ~30% ejection fraction after myocardial infarction in nude rats (Okura et al., 2010; Mishra et al., 2011), our relatively higher ejection fraction suggests the presence of only small infarcts. This is further substantiated by H&E staining of myocardial tissue sections, in which minor thinning of the left ventricular free wall was observed 1 month after infarction (Figure 6A). Owing to the mild myocardial injury model, it was not expected for cardiac function to be improved with myocardial tissue treatment.

One month after myocardial tissue implantation, the animals were euthanized, and the engineered myocardial tissues were explanted together with the myocardium for transverse tissue sectioning. The PCL scaffolds remained intact at the time of explantation and did not show signs of marked degradation. Immunofluorescence staining of iCMs and iECs using respective human-specific phenotypic markers of troponin-T and CD31 revealed no detectable cells within the explanted engineered myocardial tissues (data not shown). The engineered myocardial tissues were instead repopulated by infiltrating cells from the host, based on H&E staining (Figure 6A). Host microvessel integration within the engineered myocardial tissues was assessed by analyzing arteriole density within the scaffolds by quantifying the number of microvessel lumens with dual expression of murine-reactive CD31 and SMA (Figure 6A). Engineered myocardial tissues composed of randomly oriented scaffolds seeded with iCMs had a significantly higher arteriole density (131 ± 33/mm^2^), compared to those composed of randomly scaffolds seeded with iCMs+iECs (75 ± 6/mm^2^), or those composed of aligned scaffolds seeded with iCMs alone (76 ± 47/mm^2^) or iCMs+iECs (63 ± 7/mm^2^) (*P* < 0.05, Figure 6B). Together this data demonstrated that engineered myocardial tissues composed of randomly oriented scaffolds could significantly enhance microvessel formation in a myocardial injury model.

## Discussion

The salient findings of this study are that (1) anisotropic scaffolds can guide the organization of iCMs and iECs along the direction of the aligned microfibers *in vitro* (Figure 2); (2) in a subcutaneous transplantation model, engineered myocardial tissue composed of randomly oriented scaffolds seeded with iECs promoted the survival of iECs for up to 14 days (Figure 3), although the engineered myocardial tissues derived from aligned scaffolds preferentially guided the formation of capillaries along the direction of the microfibers (Figure 4); and (3) in a myocardial injury model, the engineered myocardial tissues composed of randomly oriented scaffolds seeded with iCMs promoted a higher arteriole density, compared to those composed of aligned scaffolds (Figure 6).

Cardiac muscle is generally comprised of parallel-aligned CMs, interspersed with parallel-aligned microvessels (Kaneko et al., 2011). Accordingly, previous studies have investigated the effect of anisotropic myocardial tissues on primary (Engelmayr et al., 2008; Bian et al., 2014; Kai et al., 2014; Lin et al., 2014) or stem cell-derived CMs (Parrag et al., 2012; Khan et al., 2015; Han et al., 2016; Li et al., 2017; Allen et al., 2019). These studies used techniques such as microsized hydrogel post arrays (Bian et al., 2014), electrospun fibrous scaffolds (Kai et al., 2014; Wanjare et al., 2017), or laser ablated materials (Bian et al., 2014) to create spatially patterned biomaterials. Although the therapeutic benefit of anisotropic myocardial tissues has been evaluated (Lin et al., 2014; Li et al., 2017), to our knowledge, this is the first study to examine the combined effect of anisotropic microfibrous scaffolds comprising human pluripotent stem cell-derived co-cultures of iCMs and iECs *in vivo*. Our results suggest that scaffold anisotropy plays a larger role than endothelial co-culture in modulating cell survival and revascularization.

To engineer myocardial tissues that mimic the ordered physiological cellular organization of the native myocardium, we employed anisotropic biomaterials to guide the assembly of the iECs and iCMs. Despite the ability of aligned microfibrous scaffolds to orient the iCMs along the direction of the aligned microfibers (Figure 2), the survival of the iCMs at 14 days after subcutaneous implantation and 28 days after epicardial transplantation was poor, suggesting that additional microenvironmental factors might be critical for the survival of iCMs *in vivo*.

In contrast to the iCMs, the retention of iECs on the scaffolds appears to be affected by both scaffold topography as well as by intercellular interactions. Based on bioluminescence imaging, iECs alone seeded on randomly oriented scaffolds had significantly higher cell survival, compared to iECs on aligned scaffolds (Figures 3A,B), suggesting a role of scaffold topography on iEC survival. However, the *in vitro* studies suggest that iEC retention on scaffolds declined when in co-culture with iCMs, independent of scaffold anisotropy. As shown in Figure 2, iECs attached at high density when cultured alone, based on CD31 expression. However, 2 days after co-culture with iCMs, the retention of CD31^+^ iECs was markedly lower on both aligned and randomly oriented scaffolds, suggesting that iCMs may impart mechanical perturbations to neighboring iECs that reduce their cell attachment over time. This is consistent with the observed decline of iEC survival *in vivo* when co-cultured with iCMs on randomly oriented scaffolds, compared to that of iECs alone (Figures 3A,B). The difference in survival between iECs and iCMs to randomly oriented scaffolds may be influenced in part by cell-specific pro-survival cues, including paracrine factors or intercellular interactions that are necessary for cell survival.

In order to support cell survival and integration, vascularization of the engineered myocardial tissue through the formation of microvasculature is critically important (Huang et al., 2018). Although we demonstrated that randomly oriented myocardial tissues were beneficial for iEC survival (Figure 3B) and host vascularization after epicardial implantation (Figure 6B), the aligned engineered tissues preferentially promoted the formation of longitudinally oriented vessels along the direction of the microfibers (Figures 4B,C), compared to randomly oriented engineered tissues. The observed differences in iEC organization *in vitro* owing to topographical patterning cues from aligned scaffolds is in agreement with previous reports (Kim et al., 2017) in which vessel-like structures derived from iECs seeded within aligned scaffolds had significantly larger branch lengths, but less branch points than those present in randomly oriented scaffolds. Mechanistically, the endothelial cells wrapped around the aligned microfibers *in vitro*, creating anisotropic network-like structures with abundant expression of paxillin, a focal adhesion protein that mediates cellular attachment to the ECM, along the primary axis of the elongated iECs. It was also reported that spatial patterning of vascular networks *in vivo* by physical constraints not only led to organized vessel formation, but also alterations in the proportion of vessel subtypes, than when compared to in the absence of spatial patterning (Chang et al., 2012). In the current study, we demonstrated that anisotropic scaffolds can guide the formation of *in vivo* microvasculature to mimic the anisotropic microvascular architecture of native myocardium (Kaneko et al., 2011). The ability to modulate the architecture of newly formed vasculature within the engineered tissues using spatially patterned scaffolds may be beneficial for vascular integration.

In the myocardial injury model, randomly oriented myocardial tissues containing iCMs alone significantly promoted arteriogenesis in an epicardial transplantation model. It is likely that randomly oriented scaffolds support more degrees of freedom in the formation of microvessels, and thereby led to a higher density of microvessels. This reasoning is supported by prior publications showing that the formation of iEC vascular networks within randomly oriented scaffolds were characterized by a greater number of vascular-like branch points, compared to those within aligned scaffolds (Kim et al., 2017). Consequently, although our findings show that vascular architecture could be tunable using anisotropic scaffolds, only the engineered myocardial tissue derived from randomly oriented scaffolds had a beneficial effect on vascularization in the myocardium. Furthermore, the small sample size and variability in ejection fraction between animals at the time of engineered tissue implantation may have precluded the possibility of observing significant improvements in cardiac function. Future studies are warranted to explore the basic mechanisms underlying scaffold-mediated cellular recruitment and vascular network formation.

Our studies also examined the effects of co-culturing iECs with iCMs on the engineered myocardial tissues. According to our results, co-cultures were not superior to monocultures with regards to iECs survival and host revascularization. These results concur with *in vitro* studies in which scaffolds seeded with iCMs alone promoted a more mature iCM phenotype and higher magnitudes of contractility, compared to scaffolds seeded with iCMs+iECs (Wanjare et al., 2017). Although it has been reported that aligned scaffolds co-cultured with primary rat adult ECs and neonatal CMs improved myocardial function after infarction, the difference in results could be due to differences in the species and phenotype of the transplanted cells (Lin et al., 2014). It is also likely that differences in the secreted pro-angiogenic milieu could favor randomly oriented scaffolds more than aligned ones.

## Conclusion

In conclusion, we demonstrated that engineered myocardial tissues derived from randomly oriented scaffolds seeded with iCMs could promote revascularization in a myocardial injury model, but engineered myocardial tissues formed from parallel-aligned scaffolds preferentially directed the formation of capillaries along the direction and plane of the microfibers. Randomly oriented scaffolds also conferred pro-survival effects to iECs, based on significantly higher cell survival upon subcutaneous transplantation. Based on these findings, we conclude that the scaffold microtopography imparts differential effects, in which randomly oriented scaffolds promote pro-survival and pro-angiogenic effects, and aligned scaffolds directed the formation of anisotropic vessels. Our findings suggest a dominant role of scaffold topography over endothelial co-culture in modulating cellular survival, vascularization, and microvessel architecture. Future studies warrant further elucidation of the signaling pathways underlying topography-mediated effects on revascularization, host cell infiltration, and cell survival.

## Data Availability

The raw data supporting the conclusions of this manuscript will be made available by the authors, without undue reservation, to any qualified researcher.

## Author Contributions

MW, CH, CA, and NH designed and carried out experiments and analyzed data. MW and NH interpreted the results. MK, HW, and YW provided technical support for animal surgeries. MW and NH wrote and organized the manuscript, with editorial input from HW.

### Conflict of Interest Statement

The authors declare that the research was conducted in the absence of any commercial or financial relationships that could be construed as a potential conflict of interest.
